# Detection of Delamination with Various Width-to-depth Ratios in Concrete Bridge Deck Using Passive IRT: Limits and Applicability

**DOI:** 10.3390/ma12233996

**Published:** 2019-12-02

**Authors:** Van Ha Mac, Quang Huy Tran, Jungwon Huh, Nhu Son Doan, Choonghyun Kang, Dongyeob Han

**Affiliations:** 1 Department of Ocean Civil Engineering, Chonnam National University, Yeosu 59626, Korea; 188456@jnu.ac.kr (V.H.M.); 188444@jnu.ac.kr (N.S.D.); hozilla@chonnam.ac.kr (D.H.); 2 Department of Civil Engineering, Nha Trang University, Khanh Hoa 57000, Vietnam; huytq@ntu.edu.vn; 3 Department of Ocean Civil Engineering, Gyeongsang National University, Tongyeong 53064, Korea; chkang@gnu.ac.kr

**Keywords:** delamination, width-to-depth ratio (WTDR), concrete bridge deck, handheld IR camera (H-IRC), UAV IR camera (UAV-IRC), passive IRT, non-destructive technique, concrete structure

## Abstract

In bridge structures, concrete decks have a higher risk of damage than other components owing to the direct impact of traffic. This study aims to develop a comprehensive system for bridge inspection using passive infrared thermography (IRT). Experiments were conducted on a concrete specimen (assumed as the surface of the bridge deck) embedded artificial delaminations with different width-to-depth ratios (WTDRs). Both professional handheld IR camera (H-IRC) and a UAV mounted with an IR camera (UAV-IRC) were employed simultaneously to capture the surface temperature of the structure. The present work indicates that the passive IRT technique with an H-IRC can be used to detect delaminations located at depths of 4 cm or less from the structure surface if the WTDRs are not lesser than 1.9 for daytime and 2.5 for nighttime when testing on a sunny day. In addition, the larger the WTDR, the higher the temperature difference can be produced, thus delaminations could be observed more clearly. Furthermore, our study suggests that the concrete bridge deck inspection using passive IRT can produce appropriate results if the inspection is performed from 10:00 to 15:00 or from 19:30 to approximately 2:00 on a sunny day. Good agreement between the results obtained from tests using H-IRC and UAV-IRC was observed, which validates the application of UAV-IRC in real structure inspection.

## 1. Introduction

**Figure 1:**
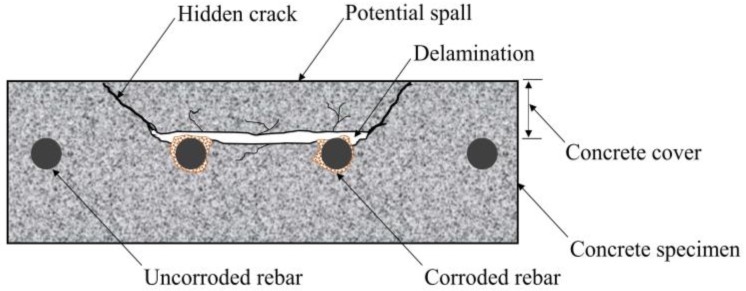
Locations of delaminations in concrete structure.

In bridge structures, compared to other components, defects occur more frequently in the concrete deck because it is directly subjected to traffic loads [1,2]. Among the various defects, delamination often develops in the concrete cover due to the corrosion effect of steel bars in reinforced concrete [2,3]. Delamination is one of the most dangerous deteriorations because it is usually invisible under visual inspection and can lead to potential spalls (Figure 1). Therefore, delaminations need to be detected as accurately as possible to maintain normal working conditions of the bridge structure.

To investigate the health of a concrete bridge deck in terms of delamination, conventional methods such as chain dragging and hammer sounding are widely used, coupled with modern non-destructive techniques (NDTs) to enhance the accuracy and quality of the inspection. The NDTs that are currently used in concrete bridge inspections include infrared thermography (IRT), ground penetrating radar, ultrasonic surface waves, impact echo, and electrical resistivity [3,4,5,6,7,8,9,10,11,12,13]. In the IRT method, there are two different approaches: passive and active [14]. In active IRT, several minutes to a few hours should be spent to inspect just small regions of structures that are not exposed directly to the sun because the structure surface must be heated by an artificial heat source [15,16,17,18,19,20,21,22,23,24,25,26,27]. On the other hand, passive IRT has been proven to be the preferred method to inspect structure components exposed directly to the sunlight [4,12,13,28,29,30,31]. The information related to potential delaminations can be extracted accurately without using any extra artificial heat source. Therefore, the concrete bridge deck is scanned more rapidly and simply using passive IRT compared to the active approach.

**Figure 2:**
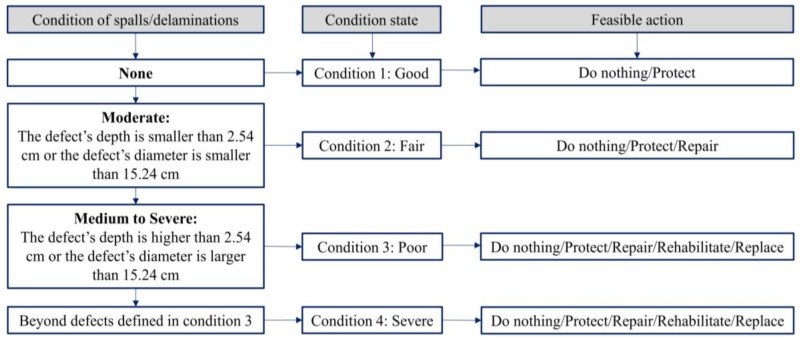
Classification of concrete bridge deck conditions required by American Association of State Highway and Transportation Officials (AASHTO) 2015.

According to the American Association of State Highway and Transportation Officials (AASHTO)-Manual for Bridge Element Inspection (2015), the condition of the concrete bridge deck is divided into four states from one to four based on the level of the severity of the damages as shown in Figure 2 [32]. If delaminations/spalls occur inside the concrete bridge deck, it means that it does not belong to the condition state 1 (good status). The condition states 2 (fair status) and 3 (poor status) correspond to the areas of moderate and severe delaminations/spalls, respectively. In the condition state 2, the delamination depth (*d*) is smaller than 2.54 cm or diameter (*w*) is less than 15.24 cm, while *d* is greater than 2.54 cm or *w* is larger than 15.24 cm in the condition state 3. When a structure is in the condition state 4 (severe status), load reduction, bridge closure, or replacement with a new structure should be performed. It is found that both the size and depth of delamination are critical indicators used to classify the health condition of concrete bridge deck. 

Moreover, although the effect of the size and depth of the delamination on the detectability of delaminations has been studied in several works, it still remains to be a controversial issue. A shallower delamination with a small size sometimes may not be detected, but a deeper delamination with a larger size may be identified. Thus, in consideration of the practical application for concrete bridge deck inspection, the employment of the passive IRT technique with various width-to-depth ratios (WTDRs, the ratios between the size and depth of delaminations) is investigated in detail in this work. This implies that the effect of the size and depth of delamination is not considered independently, like previous works, but is studied using the WTDR [1,29,30]. 

In addition, as the environment strongly affects the detectability of defects in passive IRT, suggesting a suitable inspection time is mentioned in previous researches [12,13,29,30,33,34]. However, this topic might not be studied fully, especially for defects with small size or WTDR, it therefore is one of the main tasks in the present work. In addition, the applicability of unmanned aerial vehicles (UAVs) is studied for both quantitative as well as qualitative assessment of the concrete bridge deck conditions. The UAV mounting an IR camera (UAV-IRC) is used alongside the handheld IR camera (H-IRC). The UAV-IRC is more suitable for places where the H-IRC might not be used owing to heavy and high-speed traffic. The results of the UAV-IRC would be verified by those from the H-IRC. This study determines the limits and applicability of passive IRT in detecting delamination with different WTRDs in concrete bridge deck. Hence, a comprehensive system for bridge inspection combining H-IRC and UAV is expected to be developed.

## 2. Literature Review 

Regarding the detectability of subsurface defects in concrete structures using passive IRT techniques, the main concerns growing in recent years are the effect of delamination parameters like the size, depth, and thickness, effect of the type of the infrared detector, impact of weather conditions, and effective inspection time, as well as the minimum threshold of the surface temperature difference used to define a defect as detectable or undetectable [1,29,30,35,36]. 

**Figure 3:**
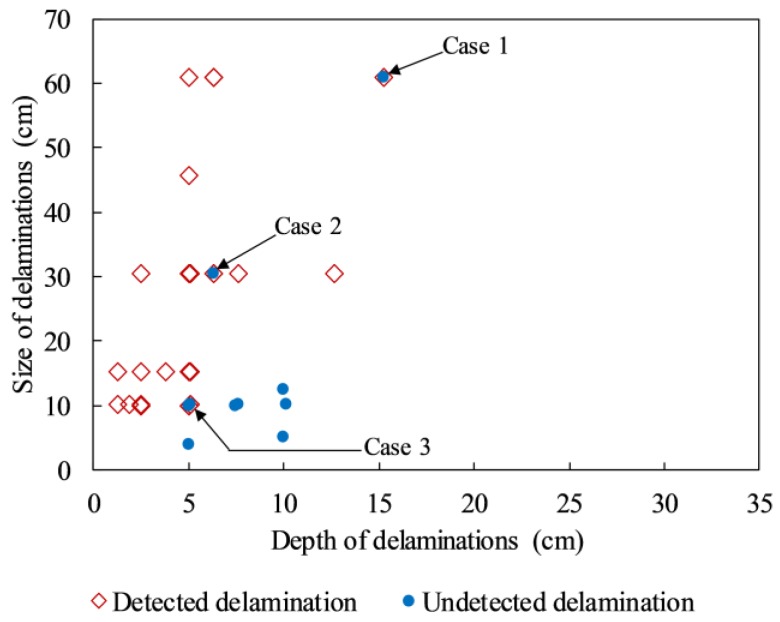
Detected and undetected delaminations summarized from the literature.

The detected depth of delaminations has been studied by researchers like Yehia et al. in 2007, Farrag et al. in 2016, Sultan et al. in 2017, and Hiasa et al. in 2018 [1,13,36,37]. Detected and undetected depths of delaminations with various sizes are depicted in Figure 3 as summarized from previous works [1,2,13,29,30,34,35,36,37,38,39]. It is noted that the results shown in Figure 3 were based on normal concrete specimens, using an uncooled IR camera, and monitored via the passive IRT approach. In Figure 3, the blue filled-in circle represents undetectable delaminations while the red unfilled-in diamond stands for detectable delaminations. 

It can be observed in Figure 3 that despite having the same WTDR, delaminations could be detected or undetected. The reason is that the experiments were performed in different environmental conditions, and the parameters of the artificial delaminations, the dimensions of concrete specimens and the specification of IR detector are not the same. For example, in the case of delaminations with the same WTDR of 4.0 shown as “Case 1” in Figure 3, one delamination could be detected while other one was not identified. The same phenomenon can be observed as pointed out in “Case 2” (WTDR = 4.8) and “Case 3” (WTDR = 2.0). Thus, the conclusions given in the previous works regarding the detected depth and size of delaminations still remain a controversial topic. Hence, further studies need to be conducted, especially in the case of heterogeneous material, such as concrete. In this work, WTDR is considered instead of the depth or size separately unlike in previous studies. 

The most effective time to conduct the concrete inspection (i.e., either daytime or nighttime) proposed in the above-mentioned studies is also inconsistent. Washer et al. in 2009 and 2010, conducted tests on a concrete specimen for approximately six months on the field [29,30]. During the test, every 10 min, the surface temperature of the specimen was monitored by an IR camera. Washer et al. recommended that the optimum times of the day for identifying delaminations at depths of 25, 51, 76, and 127 mm are after sunrise 5 h and 40 min, 6 h, 7 h and 9 h, respectively [29,30]. However, in 2018, Hiasa et al. proposed different times to perform the delamination inspection in concrete structures based on the results of the field test on four concrete specimens in which an IR camera was utilized to capture the surface temperature of the specimen from 7:00 am to 12:00 am [1]. They concluded that delaminations at depths of 5.1 and 7.6 cm could not be detected. It was suggested that delaminations at depths of 1.3 and 2.5 cm can be detected at any time except during the interchange periods of 09:00–9:30 and 15:00–16:00. The authors stated that the inspections should be conducted during nighttime because the available time during nighttime is longer than that during daytime, and the probability of the misdetection of the delaminations may be reduced. 

Furthermore, Yehia et al. (2007), Kee et al. (2012), and Gucunski et al. (2013) gave their own recommendations about the available inspection time [12,13,34]. Yehia mentioned that during the period from 10:00 to noon, delaminations with depths equal to or smaller than 5.1 cm were shown more clearly than from noon until 3 pm [13]. Focusing on deep delaminations of 6.35 and 15.24 cm, Kee et al. stated that delaminations could be detected clearly at 45 min after sunrise during the cooling cycle or after sunrise 7 h and 45 min while no delaminations were shown at 3 h and 45 min after sunrise [34]. Gucunski et al. recommended that 40 min after sunrise is a better time than around noon for the delamination inspection [12]. As reviewed above, the proposed times are completely different and this can be explained by the effects of different environmental conditions, delamination characteristics, concrete specimen dimensions, infrared detectors, and experiment setup. Hence, the determination of feasible inspection time should be studied further, especially in the case of small WTDR delamination in order to reduce the misdetection problem in the concrete bridge inspection. 

In passive IRT, the IR camera is normally kept in hand or mounted on a car to scan the concrete bridge deck. In these cases, although the lane closure is not required, the vehicle speed on the bridge is reduced and it is inconvenient in certain aspects. Recently, some researchers have utilized UAV-IRC to capture thermal images in order to conduct bridge inspection [40,41,42,43,44]. In 2017, Omar et al. used UAV-IRC to detect defects in a concrete bridge [40]. In their study, the severity of the delaminated area in the concrete bridge deck was indicated by a condition map based on their proposed objective thresholds. Khan et al. in 2015 used the UAV-IRC to collect thermal images of a mock-up bridge. As a result, potential defects in the concrete bridge deck were indicated [41]. In addition, the effect of technical data of IR camera, UAV and the overlap of individual images were considered by Vasterling and Meyer in 2013 and Gillins et al. in 2016 [43,44]. They stated that the uncooled sensor IR camera with 8–14 μm of bandwidth is a good choice for the UAV-IRC because of its lightweight. The flight altitude should be selected based on the camera resolution and separated images should be taken with a minimum overlap of 50%. In all works mentioned above, the effects of WTDRs as well as the ranges of the WTDRs in which delaminations may be detected by using UAV-IRC have not been presented. These matters will be clarified in this study and the applicability of UAV-IRC is examined by comparing its results with those of experiments from H-IRC. 

In the present work, the experiments were carried out on a concrete slab embedded artificial delaminations with various WTDRs using passive IRT. Two IR detectors with different specifications are used including an H-IRC and a UAV-IRC. To develop a comprehensive system in inspecting the bridge using both H-IRC and UAV, the aims of this study is to: (1) Examine the effect of WTDRs on the detectability of delaminations in a concrete specimen; (2) Determine the minimum WTDR of a delamination that can be detected during the daytime and nighttime; (3) Propose a suitable time for concrete bridge deck inspection using passive IRT technique; (4) Evaluate the applicability of UAV-IRC on delamination detection in concrete bridge decks. 

## 3. Fundamentals of Passive IRT

**Figure 4:**
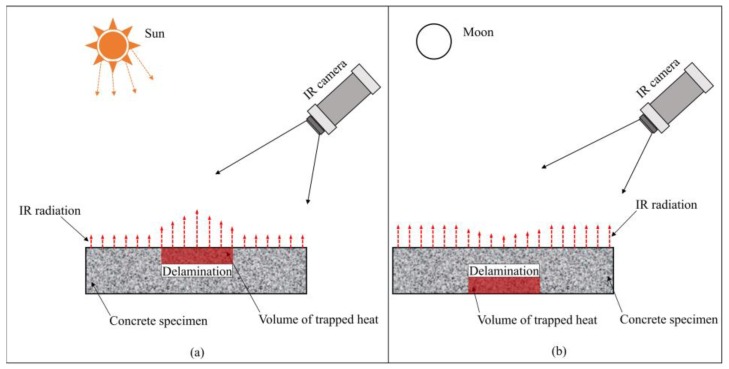
Fundamentals of defect detection using passive IRT: (**a**) during daytime; (**b**) during nighttime.

In the passive IRT technique, there are two configuration modes including transmission and reflection [28]. In transmission mode, heat sources and IR cameras are placed on two opposite sides of the specimen whereas they are placed on the same side of the specimen in the reflection mode [28]. In the present work, the reflection mode is chosen (during daytime) to detect delaminations in a concrete slab because this mode is more popular and applicable for concrete bridge deck inspection, especially in cases of bridges with great heights. The mechanism of the detectability of delaminations during both daytime and nighttime is briefly explained as shown in Figure 4. 

During the daytime, energy from the sun heats up the concrete surface. If a delamination exists inside the concrete structure, the volume of the trapped heat develops above the delamination because the thermal conductivity of air (approximately 0is much lower than that of the concrete (between 0.4 and 1.8 W/m°C), then the energy from the trap.024 W/m°C) ped heat volume turns back to the surface [1,2,45,46]. Therefore, the concrete surface above the delaminations becomes hotter than the neighborhoods during the daytime. In contrast, the heat is radiated back to the sky, thus the volume of the trapped heat is under the delamination during nighttime [1]. As a result, the concrete surface above the delamination is cooler than its surroundings during nighttime. When utilizing an IR camera to capture the thermal images at a suitable time, defects can be observed based on the difference of the surface temperature between the delaminated and surrounding areas.

**Figure 5:**
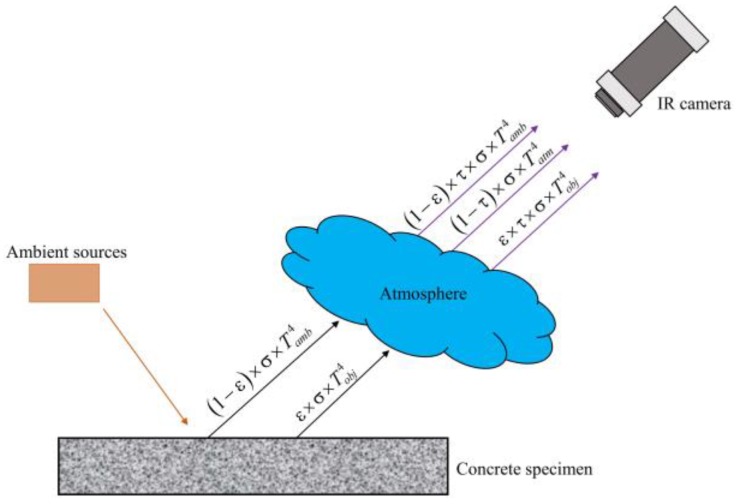
Radiation received by an infrared (IR) camera.

The total radiation (*W_tot_*) is captured by an IR camera not only from the object but also from ambient sources and the atmosphere, as shown in Equation (1) and Figure 5 [47]. The surface temperature of the object can be computed automatically by using Equation (2).

(1) $$W_{tot}=\varepsilon \times \tau \times \sigma \times T_{obj}^{4}+(1-\varepsilon )\times \tau \times \sigma \times T_{amb}^{4}+(1-\tau )\times \sigma \times T_{atm}^{4},$$

(2) $$T_{obj}={\left\{\frac{W_{tot}-(1-\varepsilon )\times \tau \times \sigma \times T_{amb}^{4}-(1-\tau )\times \sigma \times T_{atm}^{4}}{\varepsilon \times \tau \times \sigma }\right\}}^{\frac{1}{4}},$$

where $\varepsilon \times \tau \times \sigma \times T_{obj}^{4}$ is the emission from the object, $(1-\varepsilon )\times \tau \times \sigma \times T_{amb}^{4}$ is the reflected emission from ambient sources, $(1-\tau )\times \sigma \times T_{atm}^{4}$ is the emission from the atmosphere, *T_obj_* is the temperature of the object, *T_amb_* is the reflected temperature from ambient sources, *T_atm_* is the atmospheric temperature, ε is the emissivity of the object, τ is the transmittance of the atmosphere, σ is the Stefan-Boltzmann constant (σ = 5.67 × 10 − 8 W/m^2^K^4^), thereby (1 − ε) is the reflectance of the object, and (1 − τ) is the emissivity of the atmosphere. The values of ε, *T_amb_*, and *T_atm_* must be set in the camera as input data.

## 4. Experimental Work

### 4.1. Concrete Specimen Design

**Figure 6:**
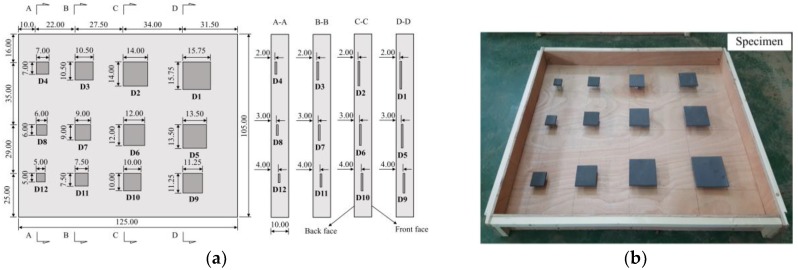
Concrete specimen with embedded imitating delaminations: (**a**) arrangement of delaminations; (**b**) formwork to cast the concrete specimen.

In this study, a concrete slab was made with dimensions 125 cm × 105 cm × 10 cm and design compression strength of 30 MPa. The coarse aggregate with maximum size of 25 mm and cement PC40 were employed to make the specimen. The proportion of water, fine aggregate and coarse aggregate to cement was 0.49, 1.48 and 1.90, respectively. The thermal diffusivity of concrete specimen was estimated as α = 1.076 cm^2^/min in our previous study [20]. Twelve squared artificial delaminations with the same thickness of 1 cm but different sizes ranging from 5.0 cm × 5.0 cm to 15.75 cm × 15.75 cm, and depths of 2, 3, and 4 cm from the back face and 5, 6, and 7 cm from front face were embedded inside the specimen that creates WTDRs from 1.0 to 7.9. Artificial delaminations were made by square pieces of polystyrene with the thermal conductivity of approximately k = 0.027 W/m°C that is expected to have very similar thermal behavior compared to the air k = 0.024 W/m°C [26,48]. In addition, artificial delaminations were glued on the stone pieces (made by concrete having same mixture ratio with the specimen) that were attached on the bottom wooden plate of formwork to create defects with exact depths as expected. Therefore, the specimen is anticipated to have similar response under heat transfer in comparison with real concrete bridge deck structure. The arrangement of delaminations and their parameters are shown in Figure 6 and Table 1.

It should be noted that if subsurface defects such as delaminations or voids develop in the concrete bridge, the air fills up inside them. These defects may achieve a similar behavior when receiving a heat flux. Therefore, the results of this study can be applied to not only delamination but also void in concrete bridge deck.

### 4.2. Experimental Setup

**Figure 7:**
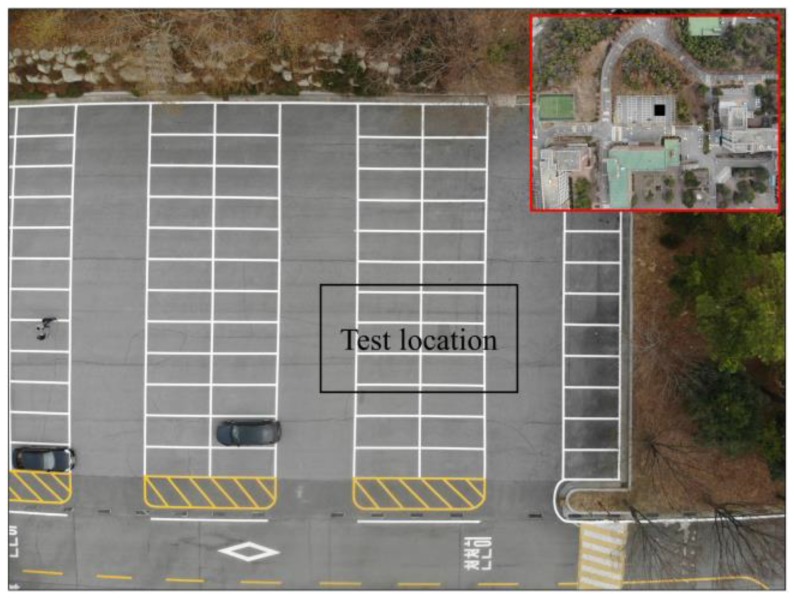
Location of the test.

**Figure 8:**
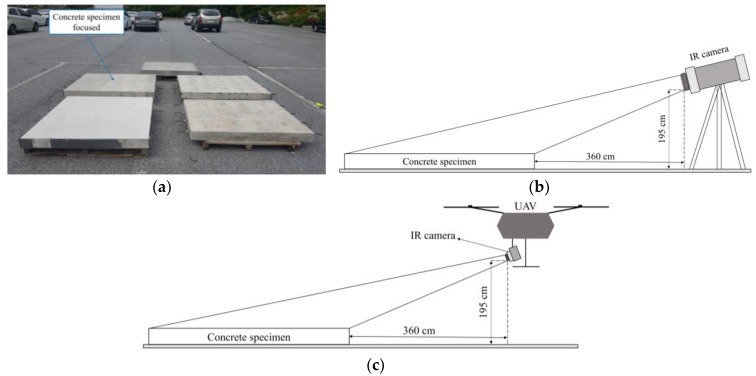
Arrangement of the experiment: (**a**) location of specimen; (**b**) with H-IRC; (**c**) with UAV-IRC.

**Figure 9:**
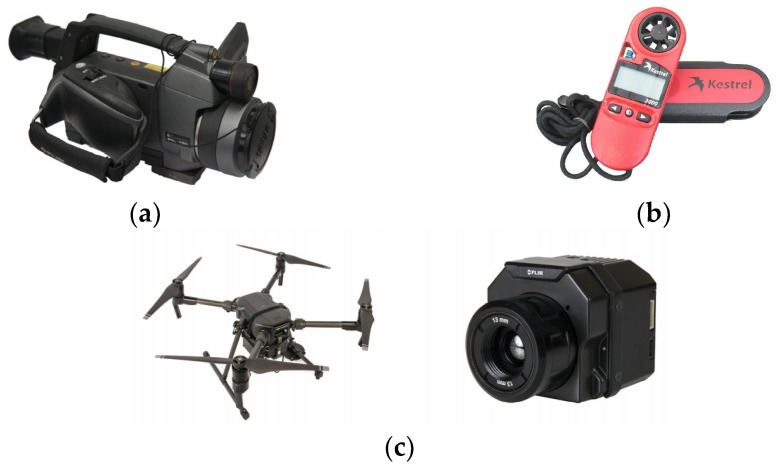
Equipment used in the experiment: (**a**) H-IRC; (**b**) Kestrel 3000; (**c**) UAV-IRC.

The passive IRT test was conducted at Chonnam National University, Republic of Korea (Figure 7 and Figure 8a). Two IR detectors were employed in this study, i.e., an H-IRC (FLIR, Wilsonville, OR, USA) and a UAV-IRC (FLIR, Wilsonville, OR, USA) (Figure 8b,c and Figure 9). The H-IRC is a long-wavelength handheld IR camera (FLIR SC660) in the 7.5–13.5 μm bandwidth [49]. This camera has a focal plane array of 640 × 480 pixels, thermal sensitivity ≤ 0.03 °C (at + 30 °C), spatial resolution (IFOV) of 0.65 mrad, field of view (FOV) of (24° × 18°)/0.3 m, and an accuracy of ±1 °C. It should be noted that long-wavelength cameras in the 7.5–13.5 μm range work well when atmospheric attenuation is involved because the atmosphere tends to act as a high-pass filter above 7.5 μm. The H-IRC is located at a height of 195 cm and the distance from the closest edge of the specimen to the camera lens is 360 cm that allows capturing the temperature of the whole specimen surface in only one thermal image (Figure 8b). Overall, the surface temperature was captured at intervals of 30 min. 

Another long-wavelength IR camera (FLIR Zenmuse XT2 - FLIR, Wilsonville, OR, USA) with the same bandwidth as that of the H-IRC is mounted on the UAV-IRC DJI M200 version [50]. The thermal sensitivity of the UAV-IRC is equal to or less than 0.05 °C, and the FOV is 57.12° × 42.44°. The technical data of the UAV is shown in Table 2 [50].

All experiments except the case of using UAV-IRC were conducted on both faces of the specimen to consider depths from 2 to 7 cm. The passive IRT test was conducted for five days. The collection time was from 6:00 to 6:00 (24 h), 8:30 to 24:00 (15 h and 30 min), 9:00 am to 21:00 pm (12 h), 8:00 to 8:00 (24 h), and 5:30 to 13:30 (8 h) on the 1st, 2nd, 3rd, 4th, and 5th days respectively. 

The scope of each experiment cycle is indicated in Table 3. The thermal images were captured only by the H-IRC during the first four days whereas both H-IRC and UAV-IRC were used on day 5. The capability of the UAV-IRC in detecting delaminations in the concrete deck is analyzed by comparing it with respect to the obtained data from the experiments using H-IRC.

The relative humidity and ambient temperature are important parameters that the camera must be fed with before capturing thermal images. Then, the surface temperature is automatically calculated by the IR camera (FLIR, Wilsonville, OR, USA) using Equation (2) in Section 3. Therefore, the ambient temperature, relative humidity as well as wind speed were measured by the Kestrel 3000 (Nielsen-Kellerman, Chester, PA, USA) before taking thermal images. The equipment used in this study is shown in Figure 9.

### 4.3. Experimental Condidion

The environmental conditions on the experimental days are summarized in Table 4. The ambient temperature, relative humidity, and wind speed are calculated as the average of the values for every three hours. Generally, the weather condition is sunny on day 1, day 3, and day 5 while day 2 is rainy and day 4 is cloudy. According to the ASTM Standards, the inspection should not be conducted when the wind speed is higher than 24 km/h [51]. The maximum wind speed on experimental days was 3.2 km/h, so all experiments were carried out under appropriate weather conditions in terms of wind speed.

As recommended in the ASTM D4788-03 Standards, the sunshine should be included because the temperature difference of 0.5 °C within a delaminated area and its neighborhood can be obtained after at least 3 h of direct sunshine [51]. Based on the data listed in Table 4, three experiment cycles including days 1, 3, and 5 are in the range while days 3 and 4 are out of the scope recommended by the ASTM Standards [51]. The non-sunny days selected in this study are for developing a comprehensive system for concrete bridge deck inspection with the combination of H-IRC and UAV-IRC under different weather conditions.

**Figure 10:**
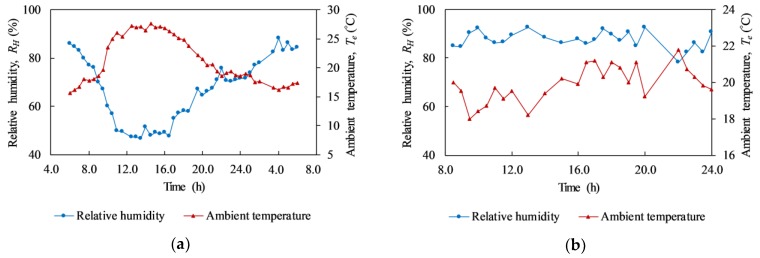
Ambient temperature and relative humidity: (**a**) day 1 (sunny day); (**b**) day 2 (rainy day).

In 2009, Washer et al. stated that the ambient temperature change is an important factor affecting the inspection outcome in passive IRT [30]. The positive ambient temperature change leads to the positive surface temperature difference above a delamination and its surrounding while it demonstrates a negative trend under the effect of negative ambient change. In addition, relative humidity change is another important factor that significantly impacts the surface temperature difference [30]. Thus, the ambient temperature and relative humidity during the test are presented in detail in Table 4 as well as Figure 10. This data is necessary for the discussion of the results as well as for the application of the study’s propositions to real structures.

The changes in relative humidity and ambient temperature during a sunny day (day 1) and rainy day (day 2) are shown in Figure 10a,b, respectively. In these figures, the red line represents relative humidity while the blue line is for ambient temperature.

On the sunny day, it can be observed that relative humidity (*R_H_*) is inversely related to the air temperature (*T_e_*); if temperature increases, the relative humidity decreases and vice versa as shown in Figure 10a. Overall, the temperature tends to go up from 6:00 and reaches the maximum value around 14:00 before falling down and towards the smallest value at 4:00 in the next day. After that, there is a small rise in the temperature from 4:00 to 6:00. In contrast, during the time between 6:00 and 14:00, the relative humidity descends dramatically. Thereafter, the relative humidity increases rapidly from 14:00 to nighttime (until 4:00). During the rainy day (Figure 10b), the tendency of both ambient temperature and relative humidity is not observed clearly. The temperature and humidity lines fluctuate significantly and there is no correlation between these two variables. 

## 5. Detectability of Delaminations Using H-IRC

### 5.1. Surface Temperature

The temperature of the concrete specimen surface is analyzed focusing on the back face with delaminations at depths equal to or less than 4 cm during both sunny day (day 1) and rainy day (day 2). It should be noted that in the experiment, sunrise appeared at 5:30 and sunset was at 18:40 on day 1. However, the sun started heating the specimen from 6:45 and stopped heating at 17:35 owing to the shadow of trees and buildings near the experiment site as shown in Figure 7. 

**Figure 11:**
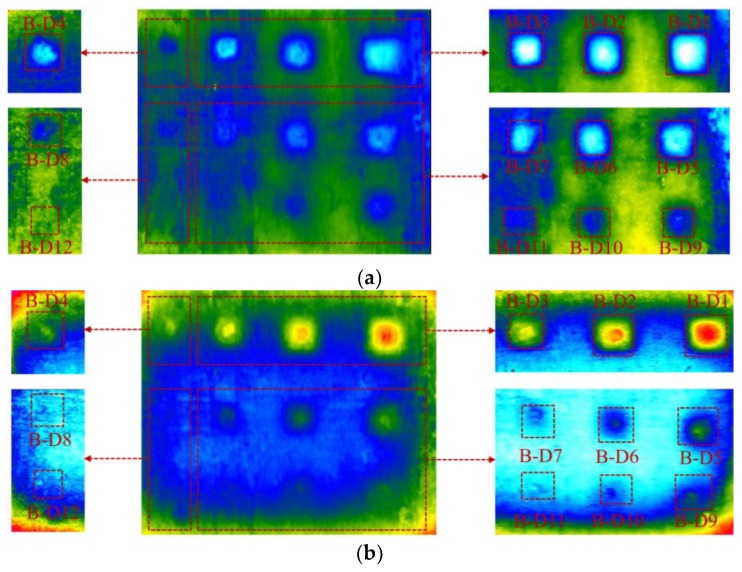
The thermal image: (**a**) at 11:00; (**b**) at 20:30.

The thermal images of the back face at 11:00 and 20:30 during the sunny day (day 1) are shown in Figure 11a,b, respectively. There are five images shown in each of the Figure 11a,b. This is because in the experiment one thermal image was captured for the entire specimen surface, and then four individual thermal images were recorded for four areas of specimen as shown by the red dashed rectangular. The reason in capturing four individual images is to reduce the temperature range of the thermal image for avoiding the misdetection of delamination that may occur if only one thermal image was recorded. There is a gap between the surface temperature above a delaminated area (*T_de_*) and its surrounding (*T_so_*). Particularly, *T_de_* is higher than *T_so_* owing to the effect of daytime heating (Figure 11a) while *T_de_* becomes cooler than *T_so_* during the nighttime (Figure 11b). Based on the temperature difference between *T_de_* and *T_so_*, delaminations can be observed on the thermal images during both the daytime and nighttime. In this section, the surface temperatures above a defect (*T_de_*) and its surrounding (*T_so_*) calculated as the mean value of surface temperatures within “delaminated area” and “sound area” respectively as presented in Section 5.2. The time when the temperature on the surface above a delamination changes from hotter to cooler in relation to its neighborhood or vice versa is called “interchange period”. The interchange period can be observed more clearly in consideration of the absolute contrast that is discussed in detail in Section 5.2. 

**Figure 12:**
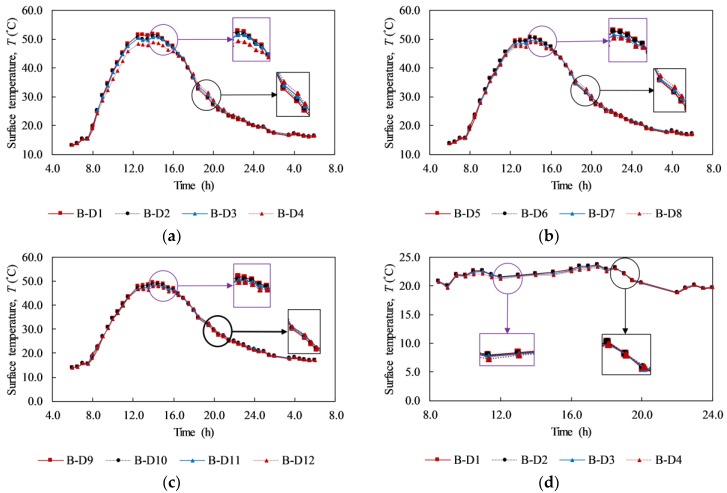
Surface temperature above delaminations: (**a**) at 2 cm depth on day 1; (**b**) at 3 cm depth on day 1; (**c**) at 4 cm depth on day 1; (**d**) at 2 cm depth on day 2.

Figure 12 shows the surface temperature (*T_de_*) within delaminated areas on day 1 and day 2. Figure 12a–c plot *T_de_* values during day 1 (the sunny day) for delaminations with depths of 2, 3, and 4 cm, respectively. It should be noted that each graph depicts delaminations that have the same depth but different sizes. Certain key phenomena can be indicated as follows: 

First, all *T_de_* value lines have the same tendency. There is a small increase of *T_de_* in the first 2 h from 6:00 before it increases considerably and reaches the peak area around noon. Around 13:30, the sky became cloudy, which causes a significant decrease in the surface temperature, as mentioned by Hiasa et al. [1]. After noon, the surface temperature rapidly drops until 22:00; however, from 22:00 on the previous day to 5:30 the next day the *T_de_* lines go down with a smaller slope. Then, the surface temperature increases slightly from 5:30 to 6:00 the next day. 

Secondly, the effect of the delamination size on the surface temperature of the specimen during daytime and nighttime can be seen clearly. Small figures are added on the graphs in Figure 12 to zoom out the difference between the *T_de_* value lines during daytime and nighttime. Overall, with the same depth, a larger delamination has a higher surface temperature than a smaller one during daytime while it becomes smaller during nighttime. For example, at 14:00, the surface temperatures are 51.66, 50.83, 50.72, and 48.81 °C corresponding to delaminations B-D1, B-D2, B-D3, and B-D4, as indicated in Figure 12. For the same set of delaminations, the observed surface temperatures are 27.00, 27.64, 28.37, and 28.90 °C at 20:30. The reason for this phenomenon is the effect of the trapped heat volume. During daytime, the trapped heat volume is developed above delamination under the heat energy from the sun. However, at the same depth, in comparison to a larger delamination, the trapped heat volume of a smaller delamination has a smaller intensity and diffuses more significantly in all directions leading to the decrease in the surface temperature [45]. During nighttime, the heat energy is radiated back into the air from the ground; thus, the volume of trapped heat is located under the delamination. Therefore, a higher surface temperature above a smaller delamination compared to a larger one is observed during nighttime. 

The surface temperature above the delaminations with different sizes at a depth of 2 cm during the rainy day is presented in Figure 12d. There is no tendency in the surface temperature change during daytime and nighttime compared to the sunny day. During the rainy day, a slightly higher surface temperature can be produced in the case of a larger delamination, but it is quite difficult to observe this as the difference is small. For instance, at 12:00 the surface temperature above delamination B-D1 (21.66 °C) has a slightly higher value (0.01 °C) than delamination B-D2 (21.65 °C). 

### 5.2. Absolute Contrast

Contrast-based methods are the simplest image processing techniques that can be applied to enhance the thermograms quality. Even though a requirement of selection of sound area is the disadvantage of these methods, they are still the most common techniques utilized to preprocess image sequences [18,28]. In this study, in order to evaluate the detectability of delaminations with different WTDRs using passive IRT, the absolute contrast (Δ*T(t)*), which is defined as the temperature difference above a defect (*T_de_(t)*) and its neighborhood (*T_so_(t)*) at the same time, as shown in Equation (3), is employed [28].

(3) $$\Delta T\left(t\right)=T_{de}\left(t\right)-T_{so}\left(t\right),$$

**Figure 13:**
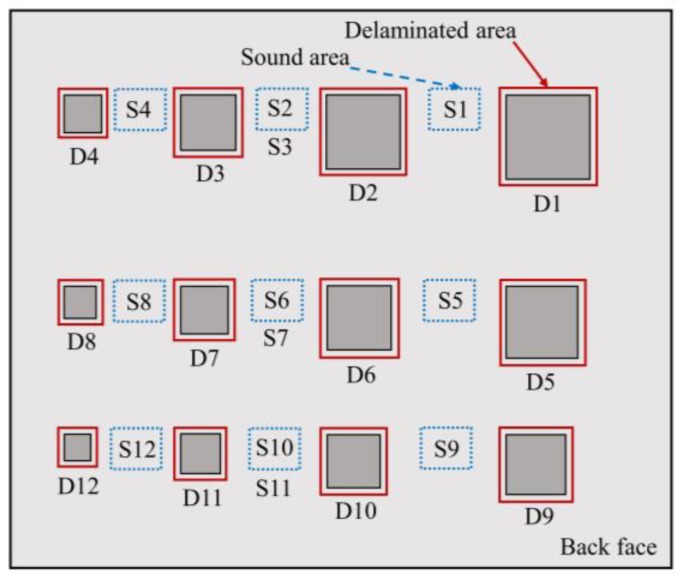
Selection of delaminated and sound areas on the structure surface.

The selection of areas of region of interest (ROI) used to compute *T_de_(t)* and *T_so_(t)* is depicted in Figure 13, where *T_de_(t)* is the average surface temperature of a ROI above a defect, called as the “delaminated area” whereas *T_so_(t)* is determined from of a ROI nearby the delamination denoted as the “sound area”. The average surface temperature is employed as mentioned by Vaghefi in 2013 that is more effective compared to only one pixel or a group of three pixels [26,52,53]. The size of delaminated areas is similar with the dimension of respective delamination while the size of all sound areas is same (around of 8 cm × 8 cm). Nine sound areas at the middle position of defects are selected for twelve delaminations. The level of upper edges of delaminated and sound area for each delamination are similar. There are three couples of delaminations at the middle region of specimen (D2 and D3, D6 and D7, D10 and D11) in which each pair refers to the same sound area to avoid the misdetection phenomenon. As per the ASTM Standards, a delamination is considered as detectable if the amplitude of the absolute contrast (Δ*T(t)*) known as “temperature difference” is 0.5 °C or higher [51]. Thus, this threshold is used in our present study. The higher the absolute contrast, the greater the certainty that delamination may appear. 

**Figure 14:**
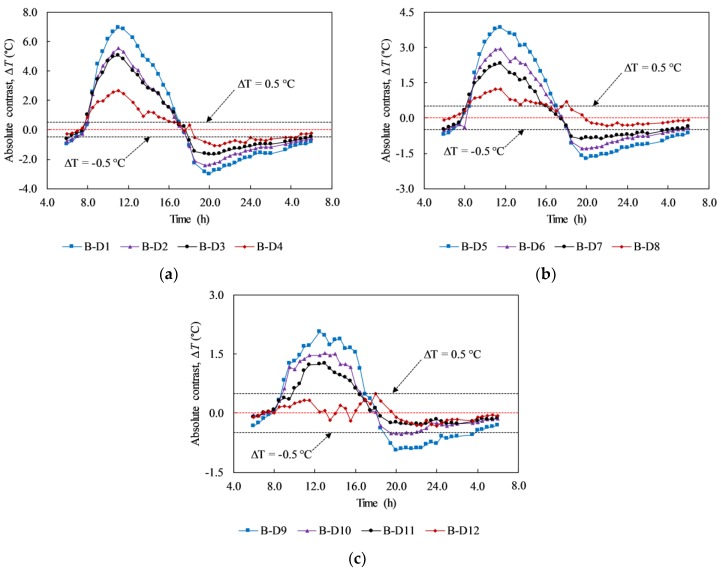
Absolute contrast of delaminations with different depths and sizes on day 1: (**a**) at the depth of 2 cm; (**b**) at the depth of 3 cm; (**c**) at the depth of 4 cm.

In the experiment, delaminations with depth of 5, 6, and 7 cm (F-D1 to F-D12) were not detected at any given time during the test (day 3 and day 4). Then, the absolute contrast is analyzed focusing on the back face that delaminations (B-D1 to B-D12) are located at depths equal to or less than 4 cm (day 1 and day 2). Figure 14 shows the absolute contrast of all delaminations during the sunny days. In each graph, delaminations with the same depth but different sizes are depicted. The absolute contrast profiles have a similar tendency in comparison with the surface temperature and ambient temperature. In general, the absolute contrast increases quickly and reaches a positive peak value around noon. After that, it reduces and attains the maximum negative value before increasing again during early morning in the next day.

Owing to the formation of the volume of trapped heat under the daytime heating effect, the surface above a delamination becomes warmer than its surrounding while it is cooler at night because of the nighttime cooling effect. It is shown in the graphs in Figure 14 that there are two interchange periods between positive values (daytime heating effect) and negative values (nighttime cooling effect) of the absolute contrast. The first interchange period occurred due to the shift from cooling effect during the nighttime to heating effect during the daytime, while the second period is caused by the change from the daytime heating effect to the nighttime cooling effect. At the interchange period, delaminations cannot be observed by the IR camera because the temperature difference between the delaminated area and sound area is small (≤0.5 °C). The interchange period lasts for approximately 2 h during both the morning time (from 6:00 to 8:00) and afternoon time (from 16:30 to 18:30) under the given experimental conditions. 

**Figure 15:**
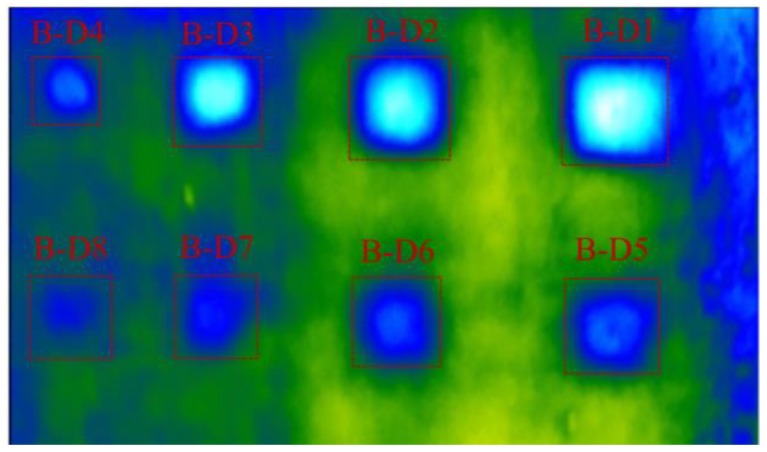
Delaminations (B-D1 to B-D8) on thermal image at 11:30 on day 1.

The effect of the size of delaminations on the temperature difference is studied as well. Under the heating effect during daytime and cooling effect during nighttime, a larger delamination produces a higher temperature difference than a smaller delamination. This phenomenon is caused by the intensity of the trapped heat volume and effect of the diffusion heat. For example, at 11:30, the temperature difference of delaminations B-D1, B-D2, B-D3, and B-D4, which have the same depth of 3 cm and different sizes of 13.5, 12.0, 9.0, and 6.0 cm, respectively, corresponds to 6.86, 5.32, 4.80, and 2.48 °C. This implies that delamination B-D1 can be observed more clearly than delaminations B-D2, B-D3, and B-D4 on the thermal image, as illustrated in Figure 15. 

**Figure 16:**
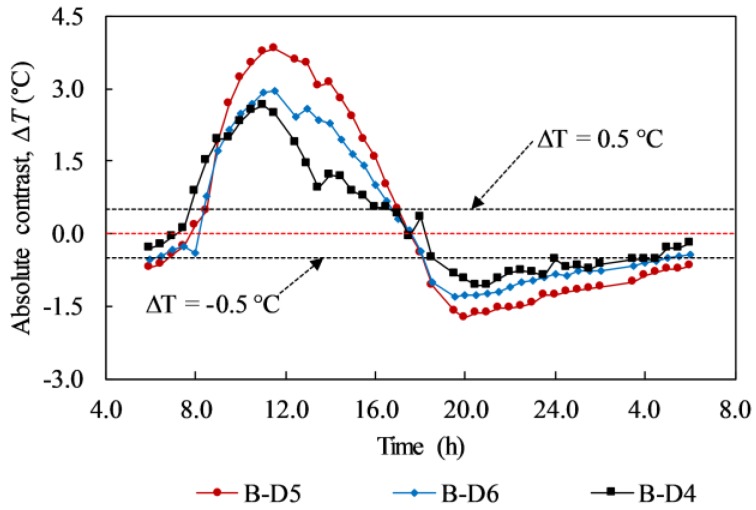
Absolute contrasts of delaminations with different WTDRs on day 1.

In terms of WTDR, it is indicated that a larger WTDR delamination can produce a higher temperature difference than a smaller WTDR one at the same depth. In addition, the conclusion can be given from the present study that a delamination, which has a larger WTDR, can produce a higher temperature difference even though it is located at greater depth than a smaller delamination as shown in Figure 16. For example, in Figure 16, delamination B-D5 and B-D6 obtained temperature differences of 3.84 °C and 2.94 °C whereas delamination B-D4 attained a temperature difference of 2.48 °C at 11:30. Moreover, the temperature differences are 1.73 °C, 1.28 °C, and 0.82 °C corresponding to delaminations B-D5, B-D6, and B-D4 at 20:00. Therefore, delamination B-D5 and B-D6 can appear more certainly than B-D4 during both the daytime and nighttime (Figure 11 and Figure 15). Furthermore, a delamination placed at a relatively greater depth experiences a time delay to obtain the maximum temperature difference than a delamination located at a relatively shallower depth from the surface as demonstrated in Figure 16. In detail, delaminations B-D5 and B-D6 achieve the maximum temperature difference at 11:30 whereas delamination B-D4 obtains the maximum temperature difference at 11:00. 

**Figure 17:**
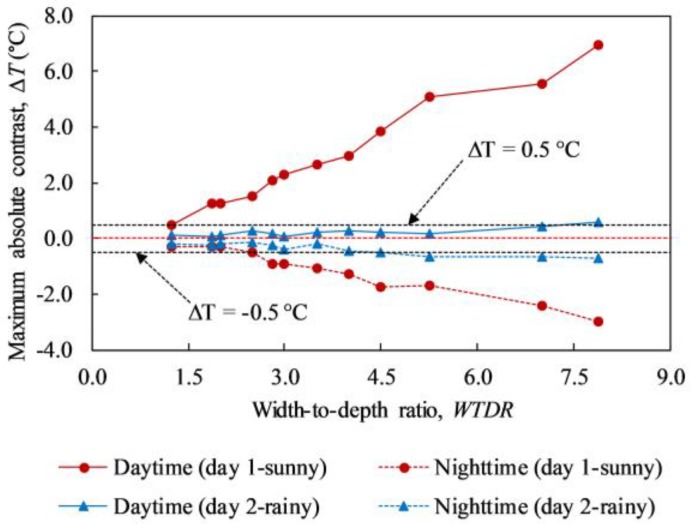
Maximum absolute contrast of delaminations during daytime and nighttime on both days 1 and 2.

The maximum absolute contrasts of all delaminations composed in this study are graphically depicted in Figure 17. The red solid and red dashed line represent the maximum absolute contrast during daytime and nighttime on a sunny day, respectively. It can be concluded that a higher WTDR delamination obtains a greater maximum temperature difference than a smaller WTDR delamination on a sunny day. The maximum temperature difference rises from 0.49 °C to 6.95 °C (positive value) and from 0.32 °C to 3.00 °C (negative value) corresponding to the daytime and nighttime when the WTDR increases from 1.25 to 7.9. However, under the effect of rain, the above-mentioned trend is not observed clearly although the maximum temperature difference may tend go up with the rise of WTDR, as indicated in Figure 17. In addition, almost all delaminations obtain the maximum temperature difference smaller than 0.5 °C. Thus, it is recommended that the concrete bridge deck inspection must not be conducted while it is raining or after rain.

### 5.3. Detection of Delamination

**Figure 18:**
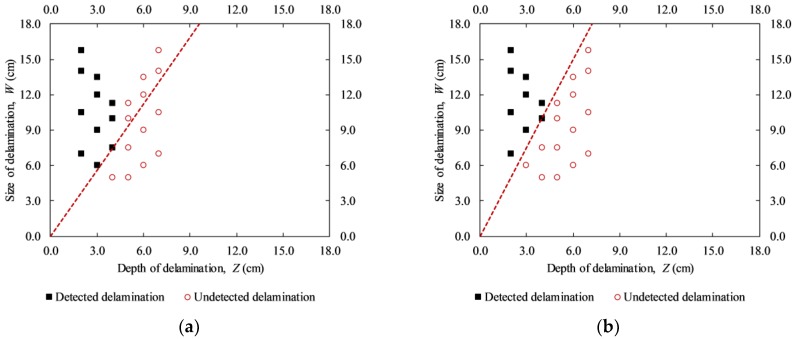
Detected and undetected delaminations on the sunny day (day 1 and day 3): (**a**) daytime; (**b**) nighttime.

The detectability of delaminations during both daytime and nighttime on the sunny day are pointed out in Figure 18. The black filled-in squares and red unfilled-in circles represent detected and undetected delaminations respectively. It should be noted that delaminations with depths equal to or higher than 5.0 cm and the given sizes in this study were not detected. Thus, the red dashed line is used to divide the graph into two regions: detectable and undetectable regions, considering delaminations with depths equal to or smaller than 4.0 cm. 

In Figure 18, the horizontal axis shows the delamination depth while the vertical axis is for the delamination size; hence the detectability of delaminations can be indicated in terms of WTDR. Certain conclusions can be given based on Figure 18 and information presented under Section 5.2 as follows.

For delaminations with depths higher than 4.0 cm (F-D1 to F-D12) on both sunny and cloudy days (day 3 and day 4): All delaminations could not be detected at any given time of the experiment period. This implies that it is impossible to identify delaminations with depths deeper than 4.0 cm and WTDRs equal to or smaller than 2.25 under the conditions of this study. 

For delaminations with depths equal to or smaller than 4.0 cm (B-D1 to B-D12) on the sunny day (day 1): In the daytime, the dashed line has a slope of 1.9 that implies a delamination whose size is not smaller than 1.9 times its depth (WTDR ≥ 1.9) may be detected (Figure 18a). However, a delamination with a WTDR of 1.9 could not be identified during the nighttime (Figure 18b). The WTDR during the nighttime should be at least 2.5 for a delamination to become detectable (the dashed line with a slope of 2.5). 

For delaminations with depths equal to or smaller than 4.0 cm (B-D1 to B-D12) on the rainy day (day 2), delamination could hardly be identified.

### 5.4. Effective Time for Delamination Detection

In passive IRT, the favorable time in capturing thermal images is a very important factor in order to be applied effectively to real structure inspections. On the rainy day (day 2), almost all delaminations could not be detected owing to the small temperature differences. Therefore, the concrete bridge deck inspection should be carried out on a sunny day. 

**Figure 19:**
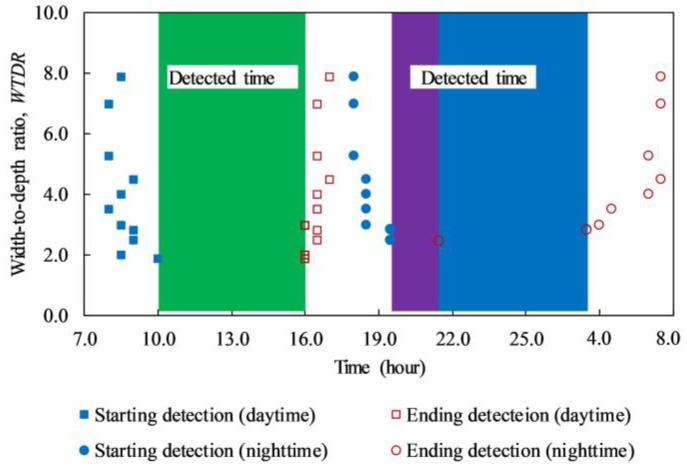
Feasible time on a sunny day to detect delamination in concrete bridge deck.

The detected time of delaminations from the back face on days 1 and 3 is graphically depicted in Figure 19. The blue filled-in square, blue filled-in circle, red unfilled-in square, and red unfilled-in circle represent the first time during daytime, first time during nighttime, last time during daytime, and last time during nighttime when the delaminations can be detected respectively. Under the effect of the heating cycle during the daytime and cooling cycle during the nighttime, there are two ranges of detectable time when the defects with depths equal to or smaller than 4.0 cm may be detected.

Under the effect of the heating cycle (daytime): The structure inspection should be conducted from 10:00 until 16:00 (6 h) to detect delaminations with WTDRs equal to or higher than 1.9 as shown by the green rectangular in Figure 19. If a delamination with a WTDR of 1.9 is eliminated, the feasible time is from 9:00 to 16:00 (7 h) to identify delamination with the WTDRs equal to or higher than 2.0. However, the inspection of the structure should be carried out from 10:00 to 15:00 (5 h) such that significantly high temperature difference can be obtained, and a clearer observation of delamination can be achieved as shown in Figure 11 and Figure 15.

Under the effect of the cooling cycle (nighttime): If the structure is inspected from 19:30 to 3:30 (8 h), delaminations with the WTDR equal to or higher than 2.8 can be identified. However, in the case of delaminations with WTDRs from 2.5 to 2.8, the suitable collection time is shortened as the period from 19:30 to 21:30 (2 h). In addition, the temperature difference of delaminations achieves a significantly high value from 19:30 to around 2:00 (6.5 h), the structure inspection therefore should be conducted during this time to achieve good results. 

In fact, the detected time of the delaminations lasts significantly during both daytime and nighttime. Therefore, the concrete bridge deck inspection can be conducted effectively throughout the day. However, during the daytime, the WTDRs of delaminations that can be detected using the passive IRT technique are smaller in comparison with the nighttime. Thus, daytime is a better choice for concrete bridge inspection than nighttime even if the delaminations with small WTDRs (≤ 2.5) are considered in the inspection. In this case, the limitation of WTDR is approximately 1.9. Conversely, if the inspection focuses only on delamination with WTDRs of 2.8 or higher, the nighttime offers a more efficient selection because of its longer detected time compared to daytime.

## 6. Detectability of Delaminations Using UAV-IRC

In the case of using the UAV-IRC, the detectability of delamination is also evaluated by using the absolute contrast measurement that is calculated from Equation (3). The temperature difference is the amplitude of the absolute contrast. 

**Figure 20:**
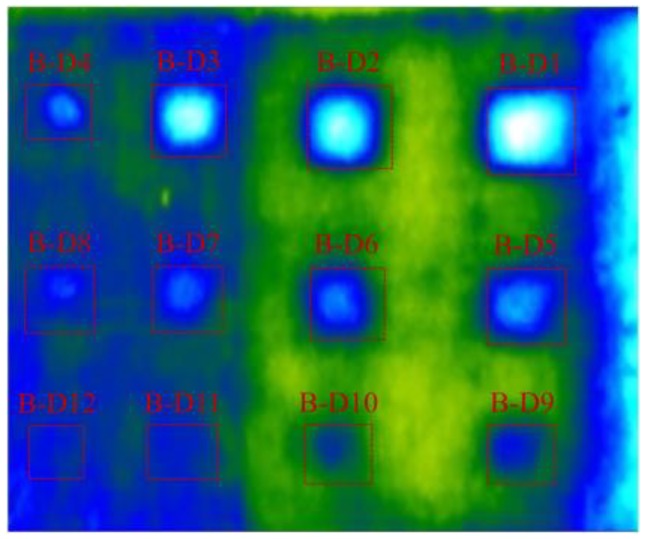
Thermal image of specimen surface using UAV-IRC at 11:30.

Figure 20 shows the thermal image at 11:30 by using UAV-IRC. The temperature difference of delaminations (B-D1 to B-D11) ranges from 5.73 to 0.54 °C that is higher than 0.5 °C, while it is 0.4 °C for the delamination B-D12. The largest WTDR delamination obtains the highest temperature difference (5.73 °C) whereas the smallest temperature difference is achieved in the case of the least WTDR delamination (0.4 °C). Therefore, all the given delaminations can be seen on the thermal images captured by using UAV-IRC except for delamination B-D12. Hence, it can be concluded that the UAV-IRC might detect delaminations at the depth equal to or lower than 4 cm and with WTDRs not smaller than 1.9 during daytime on the sunny day. However, it should be noted that delamination B-D11 (WTDR = 1.9) is still quite difficult to be recognized as shown in Figure 20.

**Figure 21:**
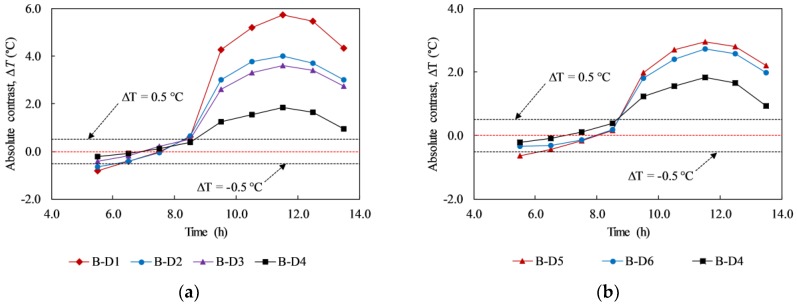
Absolute contrast of delaminations with different WTDRs on day 5 from UAV-IRC: (**a**) at same depth of 2 cm; (**b**) at different depths of 2 and 3 cm.

Figure 21 shows the absolute contrast of delaminations having different WTDRs on day 5. Particularly, Figure 21a shows the case of defects at the same depth of 2 cm (B-D1, B-D2, B-D3, and B-D4) while Figure 21b depicts defects at depths of 2 (B-D4) and 3 cm (B-D5 and B-D6). The WTDRs of delaminations B-D1, B-D2, B-D3, and B-D4 are 7.9, 7.0, 5.25, and 3.5, respectively, whereas the WTDR is 4.5 and 4.0 corresponding to delaminations B-D5 and B-D6. The same tendency can be observed in comparison with the results obtained from the H-IRC that a larger delamination WTDR achieves higher temperature difference than a smaller one, although it is located at the same depth or further from the surface of the structure. For example, in the case of different WTDRs at the same depth of 2 cm, at 10:30, the temperature differences are 5.22, 3.78, 3.30, and 1.55 °C corresponding to delaminations B-D1, B-D2, B-D3, and B-D4. On considering delaminations with different WTDRs and depths, delamination B-D5, B-D6, and B-D4 obtained the temperature difference of 2.70, 2.40, and 1.75 °C at 10:30, respectively. Consequently, delamination B-D5 and B-D6 can appear more clearly than B-D4.

The interchange period occurred in the period from 06:30 to 08:30 on day 5 that is very similar to the results from experiments using H-IRC on day 1. It should be noted that the experiments on both days 1 and 5 focus on the back face and the overall weather conditions are sunny during these days. There is only 30 min of the delay time of the interchange period between day 5 and day 1. It might be caused by the difference between the interval in capturing thermal images that are 30 min and 1 h in the case of day 1 and day 5, respectively.

**Figure 22:**
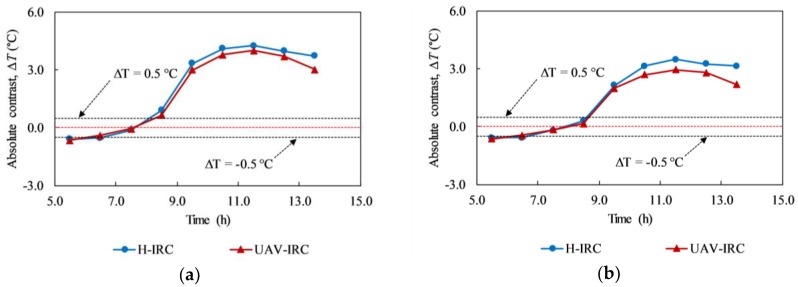
Comparison between the absolute contrast captured by H-IRC and UAV-IRC on a sunny day (day 5): (**a**) delamination B-D2; (**b**) delamination B-D5.

Figure 22 shows the absolute contrast of delamination B-D2 (depth of 2 cm) and B-D5 (depth of 3 cm) based on the results of experiments using UAV-IRC and H-IRC on day 5. In Figure 22a,b, the blue line represents H-IRC while the red line stands for UAV-IRC. At early hours in the morning, the variance of temperature difference between UAV-IRC and H-IRC, known as DIF is much smaller than at other times because the surface temperature in the early morning is more stable than at noon as well as the afternoon, and that time is nearby the interchange period. For example, the DIF is 0.05 °C at 5:30 while it is 0.29 °C at 12:30 in the case of delamination B-D2. Furthermore, at 11:30, both H-IRC and UAV-IRC obtained the maximum temperature differences. These maximum values are 3.48 °C and 2.95 °C for B-D5 corresponding to the outcome from H-IRC and UAV-IRC. In addition, it is shown that larger the delamination depth, higher the DIF obtained except for the observation at 9:30 am. For instance, DIFs are 0.29 °C and 0.44 °C at 12:30 corresponding to delamination B-D2 and B-D5. 

Moreover, as shown in Figure 22a,b, both lines show the same tendency, but the red line is located more closely to zero axis compared to the blue line. This can be explained by the effect of the wind from the fans of UAV and the smaller thermal sensitivity of IR camera mounted on the UAV in comparison with H-IRC leading to the decrease in absolute contrast in case of UAV-IRC. However, the gap between the two lines is not significant, which implies that there is a good agreement between the results obtained from experiments using UAV-IRC and H-IRC. Consequently, it can be stated that under the given environmental conditions, using UAV-IRC can give appropriate results. The effective application of UAV-IRC in the defect detection of concrete bridge decks is confirmed. 

## 7. Conclusions

In this study, an effort was made to consider the possibility of simultaneously using the results from the handheld IR camera (H-IRC) and the IR camera hanged on a UAV (UAV-IRC). From the results presented in this work under given experimental conditions and processing method, the following conclusions are obtained:

For delaminations at the depths of 4.0 cm or lesser, the larger the WTDRs of the delaminations, the higher is the temperature difference produced on the thermal image, which implies that there is a strong correlation between the size and detected depth of delamination.

The minimum WTDR of a delamination that can be detected by using H-IRC was determined in this study. Delaminations with WTDRs of 1.9 or larger located at depths of 4.0 cm or lesser from the structure surface may be identified during the daytime on a sunny day, whereas during the nighttime of a sunny day, only delaminations with WTDRs of 2.5 or larger can be discovered. However, if the delamination depth is 5.0 cm or higher and its WTDR is under 2.25, it may not be noticeable on the thermal images.

Passive IRT must be applied to scan the concrete bridge deck on a sunny day because adequate heat energy is provided that helps to extract accurately the delamination occurrence. Generally, the optimal time to inspect the concrete bridge deck is from 10:00 to 15:00 and from 19:30 to around 2:00, respectively, during the daytime and nighttime on a sunny day. 

Although the absolute contrast produced in the case of UAV-IRC is slightly smaller than H-IRC, UAV-IRC also can be employed to detect delaminations with the depths equal to or less than 4.0 cm and WTDRs of 1.9 or larger during the daytime of a sunny day. Thus, it is proven that UAV-IRC in the passive IRT technique is a feasible solution that can be utilized separately or simultaneously with H-IRC to discover the delamination with a shorter inspection time compared to H-IRC in the concrete bridge deck.

## Figures and Tables

**Table 1:** Characteristics of artificial delaminations inside the concrete specimen.

Delamination	Sizes (cm × cm × cm)	From Back Face			From Front Face		
		Depth (cm)	WTDR	Denotation	Depth (cm)	WTDR	Denotation
D1	15.75 × 15.75 × 1.0	2	7.9	B-D1	7	2.25	F-D1
D2	14.0 × 14.0 × 1.0	2	7.0	B-D2	7	2.0	F-D2
D3	10.5 × 10.5 × 1.0	2	5.25	B-D3	7	1.5	F-D3
D4	7.0 × 7.0 × 1.0	2	3.5	B-D4	7	1.0	F-D4
D5	13.5 × 13.5 × 1.0	3	4.5	B-D5	6	2.25	F-D5
D6	12.0 × 12.0 × 1.0	3	4.0	B-D6	6	2.0	F-D6
D7	9.0 × 9.0 × 1.0	3	3.0	B-D7	6	1.5	F-D7
D8	6.0 × 6.0 × 1.0	3	2.0	B-D8	6	1.0	F-D8
D9	11.25 × 11.25 × 1.0	4	2.8	B-D9	5	2.25	F-D9
D10	10.0 × 10.0 × 1.0	4	2.5	B-D10	5	2.0	F-D10
D11	7.5 × 7.5 × 1.0	4	1.9	B-D11	5	1.5	F-D11
D12	5.0 × 5.0 × 1.0	4	1.25	B-D12	5	1.0	F-D12

**Table 2:** Main technical data of the DJI M200 version UAV.

Items	Parameters
Dimensions (unfolded)	887 mm × 880 mm × 378 mm
Self-weight (with two TB50 batteries)	~3.80 kg
Max payload (with two TB50 batteries)	~2.34 kg
Max angular velocity	Pitch: 300 °/s; Yaw: 150 °/s
Max speed	60.8 km/h
Max wind resistance	12 m/s
Max flight time	27 min

**Table 3:** Time cycle for the experiment.

Day	Duration (h)	Face Exposed Directly to the Sun	Camera Used
Day 1	6:00–24:00	Back face	H-IRC
	0:00–6:00	Back face	
Day 2	8:30–24:00	Back face	
Day 3	9:00–21:00	Front face	
Day 4	8:00–24:00	Front face	
	0:00–8:00	Front face	
Day 5	5:30–13:30	Back face	H-IRC and UAV-IRC

**Table 4:** Weather conditions during the experiments.

Day	Time (h)	Average of Ambient Temperature (°C)	Average of Relative Humidity (%)	Average of Wind Speed (km/h)	Weather Condition	Overall Condition
Day 1	6:00–9:00	17.2	79.5	0.8	Sunny, partly cloudy	Sunny day
	9:00–12:00	23.0	58.8	0.6	Sunny, clear sky	
	12:00–15:00	27.0	48.3	2.2	Sunny, partly cloudy	
	15:00–18:00	26.1	52.1	1.2	Sunny, clear sky	
	18:00–21:00	22.2	63.5	0.6	Clear sky	
	21:00–24:00	19.2	71.1	0.4	Clear sky	
	0:00–3:00	18.3	74.0	0.1	Clear sky	
	3:00–6:00	16.7	84.6	0.0	Clear sky	
Day 2	8:30–9:00	19.8	84.9	0.4	Moderate rain	Rainy day
	9:00–12:00	19.0	88.2	1.2	Moderate rain	
	12:00–15:00	19.3	89.2	1.0	Light rain	
	15:00–18:00	20.6	88.2	0.3	Cloudy	
	18:00–21:00	20.4	89.0	0.4	Heavy rain	
	21:00–24:00	20.4	83.9	0.8	Light rain	
Day 3	9:00–12:00	28.3	66.6	0.4	Sunny, clear sky	Sunny day
	12:00–15:00	30.1	59.8	0.9	Sunny, clear sky	
	15:00–18:00	28.1	61.6	0.9	Sunny, partly cloudy	
	18:00–21:00	22.8	74.8	0.6	Partly cloudy	
Day 4	8:00–9:00	20.6	83.5	0.8	Cloudy	Cloudy day
	9:00–12:00	19.0	89.9	1.2	Cloudy	
	12:00–15:00	20.3	83.5	1.5	Cloudy	
	15:00–18:00	23.4	73.8	1.0	Mostly cloudy	
	18:00–21:00	20.5	78.2	0.4	Mostly cloudy	
	21:00–24:00	18.5	90.5	0.0	Cloudy	
	0:00–3:00	18.9	89.5	0.0	Cloudy	
	3:00–6:00	19.4	90.0	0.0	Cloudy	
	6:00–8:00	21.9	76.3	0.4	Cloudy	
Day 5	5:30–8:00	13.8	67.3	3.2	Sunny, partly cloudy	Sunny day
	8:00–11:00	16.7	56.2	2.8	Sunny, clear sky	
	11:00–13:30	21.9	41.8	2.9	Sunny, clear sky	
